# Fruit and Vegetable Consumption and the Risk of Bone Fracture: A Grading of Recommendations, Assessment, Development, and Evaluations (GRADE)‐Assessed Systematic Review and Dose–Response Meta‐Analysis

**DOI:** 10.1002/jbm4.10840

**Published:** 2023-11-10

**Authors:** Sheida Zeraattalab‐Motlagh, Seyed Mojtaba Ghoreishy, Arman Arab, Sara Mahmoodi, Amirhossein Hemmati, Hamed Mohammadi

**Affiliations:** ^1^ Department of Community Nutrition, School of Nutritional Sciences and Dietetics Tehran University of Medical Sciences Tehran Iran; ^2^ Department of Nutrition, School of Public Health Iran University of Medical Sciences Tehran Iran; ^3^ Student Research Committee, School of Public Health Iran University of Medical Sciences Tehran Iran; ^4^ Division of Sleep Medicine Harvard Medical School Boston Massachusetts USA; ^5^ Medical Chronobiology Program, Division of Sleep and Circadian Disorders Departments of Medicine and Neurology, Brigham and Women's Hospital Boston Massachusetts USA; ^6^ Department of Clinical Nutrition, School of Nutritional Sciences and Dietetics Tehran University of Medical Sciences Tehran Iran

**Keywords:** COHORT, FRACTURE, FRUIT, META‐ANALYSIS, VEGETABLES

## Abstract

Researchers have examined the link between consuming fruit and vegetables and the incidence of fractures for many years. Nevertheless, their findings have been unclear. Furthermore, the dose‐dependent relationship has not been examined, and the level of certainty in the evidence was not evaluated. We carried out a dose‐dependent meta‐analysis examining the relation between fruit and vegetables intake and fracture incidence. PubMed, Web of Sciences, and Scopus were searched until April 2023 for cohort studies evaluating the relation between fruit and vegetables and fracture incidence. Summary relative risks (RRs) were computed from complied data by applying random effects analysis. To examine the level of evidence, we utilized the approach called the Grading of Recommendations, Assessment, Development, and Evaluations (GRADE). Ten cohort studies comprising 511,716 individuals were entered. There was a nonsignificant relation between fruit and vegetables, as well as only fruit intake and any fracture risk. In contrast, high versus low analysis presented that vegetables consumption was linked to a 16% decrease in any type of fracture incidence (RR 0.84; 95% confidence interval [CI], 0.75 to 0.95; *I* ^2^ = 83.1%; *n* = 6). Also, per one serving/day (200 g/day) increments in vegetables consumption, there was a 14% decline in the fracture risk (RR 0.86; 95% CI, 0.77 to 0.97; *I* ^2^ = 84.7%; *n* = 5; GRADE = moderate). With moderate certainty, a greater consumption of only vegetables, but not total fruit and vegetables or only fruit, might reduce the risk of fracture. These associations were also evident in dose–response analysis. Large intervention trials are demanded to approve our findings. © 2023 The Authors. *JBMR Plus* published by Wiley Periodicals LLC on behalf of American Society for Bone and Mineral Research.

## Graphical abstract

In the present study, we found that with moderate certainty, a greater consumption of only vegetables, but not total fruit and vegetables or only fruit, might reduce the risk of fracture. Per one serving/day increment in intake of vegetables, there is a 14% decrease in fracture risk. Large intervention trials are demanded to approve our findings

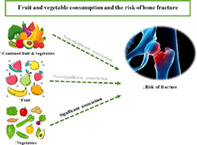

## Introduction

Fracture of bones represents a major public health issue among the elderly population worldwide,^[^ ^1^, ^2^ ^]^ with over 8.9 million osteoporotic fractures occurring every year.^[^ ^3^ ^]^ Many individuals suffer from this disorder, leading to permanent damage and severe health complications, including elevated mortality and diminished health‐related quality of life.^[^ ^4^, ^5^ ^]^ The cost of incident fractures on the economy each year is significant.^[^ ^6^ ^]^ For instance, the cost of medical care for hip fractures is predicted to be US $10,075, and the global average of social care costs following 1 year is US $43,669.^[^ ^7^ ^]^

Lifestyle and behavioral factors are linked to fracture risk,^[^ ^8^, ^9^ ^]^ of which diet is considered one of the main reasons for bone quality and fracture risk.^[^ ^10^ ^]^ Consuming more fruit and vegetables, which are inevitable parts of healthy diets, is related to lower bone loss,^[^ ^11^ ^]^ as well as bone turnover,^[^ ^12^ ^]^ and is also linked with increased bone density.^[^ ^13^, ^14^ ^]^ Consuming fruit and vegetables has a favorable impact not only on the bone but also on skeleton health, which is assumed to be related to certain elements of these foods, such as carotenoids,^[^ ^15^ ^]^ minerals (potassium, magnesium, zinc, etc.),^[^ ^16^ ^]^ vitamins K and E, ascorbic acid,^[^ ^17^, ^18^, ^19^, ^20^ ^]^ and other antioxidizing elements like flavonoids and polyphenols,^[^ ^21^ ^]^ as well as phytoestrogens.^[^ ^22^ ^]^

A cohort study conducted among Swedish adults indicated that contrary to subjects who consumed five servings/day of fruit and vegetables, those with no consumption had an 88% greater hip fracture risk.^[^ ^23^ ^]^ This finding has been confirmed by the Consortium on Health and Aging: Network of Cohorts in Europe and the United States (CHANCES) project, which compiled data from 14 cohort studies conducted in the United States, as well as Europe, which reported a 39% elevated incidence of hip fractures among those who ate one or fewer servings/day of fruit and vegetables in contrast to those who ate moderate amounts (>3 and ≤5 servings/day).^[^ ^24^ ^]^ Previously, Lu et al.^[^ ^25^ ^]^ reported in a review that greater consumption of vegetables although not fruit, was related to a lower hip fracture incidence. In contrast, a review of intervention trials and cohorts demonstrated a relationship between increasing every 200 g of fruit and vegetables daily and reducing fracture incidence.^[^ ^26^ ^]^ Previous papers mainly focused on hip fractures, and they did not provide enough information regarding the fracture risk of other sites, as well as examining the evidence certainty. Moreover, the relationship between each certain amount (per 1 serving/day) increment of fruit and vegetables intake and any fracture incidence has not been evaluated yet. Therefore, we intended to carry out a systematic review and dose‐dependent analysis of prospective cohort studies in adults to confer a quantitative estimate of the relation of fruit and vegetables with overall fracture risk. We hypothesized that greater fruit and vegetables consumption could improve fracture risk.

## Methods

We implemented a meta‐analysis in accordance with the Cochrane Handbook for Systematic Reviews of Interventions,^[^ ^27^ ^]^ as well as Grading of Recommendations, Assessment, Development, and Evaluations (GRADE).^[^ ^28^ ^]^ A protocol for our systematic review has been recorded in PROSPERO with the registration number CRD42023431027.

### Systematic search

Following the Preferred Reporting Items for Systematic Reviews and Meta‐Analyses (PRISMA), we implemented this meta‐analysis.^[^ ^29^ ^]^ A literature review was implemented on databases of PubMed, Web of Science, and Scopus from the earliest available date up to April 2023, regardless of language restrictions or time limitations, using search terms including [“Fruit” OR “vegetable” OR “fruits” OR “vegetables” OR “fruit*”] AND [“bone” OR “bone fracture” OR “fracture” OR “osteoporotic fracture”] (Table S1). Disagreements were addressed by a third investigator (HM). Additionally, the references of reviews examining fruit and vegetables consumption and fracture incidence were screened to prevent any missing. Two reviewers (SZM and SM) separately screened the titles or abstracts, as well as full‐text of individual cohort studies. If necessary, the third investigator (HM) was consulted to resolve disagreements.

### Inclusion and exclusion criteria

To include relevant papers, they need to meet the outlined subsequent criteria: (i) designed as a prospective study; (ii) examined the association of fruit and vegetables consumption with fracture incidence in general adults (aged at least 18 year); and (iii) given enough details about effect estimates (relative risk [RR], hazard ratio [HR], rate or risk ratios or odds ratio [OR], and corresponding 95% confidence intervals [95%CIs]). We chose prospective cohort studies that met the abovementioned criteria and provided comprehensive documents to conduct dose–response analysis. This included studies that displayed exposures as groups and provided sufficient information across those groups. Otherwise, we selected studies with the largest number of total individuals. We excluded letters, publications with identical participants, reviews, intervention trials, abstracts, cross‐sectional or case–control papers, and published papers conducted on children, as well as adolescents.

### Data extraction

Two of us (SM and SMG) pulled out the required data from the eligible studies. In the condition of a disagreement, a discussion with the third reviewer (HM) was conducted if appropriate. The following information was extracted from the cohort studies: last name of the first author, study location and name, publication time, individual sex and age, length of the study, sample size comprising both participants and fracture events, type of exposure and fracture, comparison, effect estimates (RR, OR, or HR with 95%CI), methods that were applied for measuring exposure and outcome and controlled variables. When several adjustment models were reported, the fully adjusted model was used for this review.

### Risk of bias (quality) assessment

To assess individual cohort study quality, we utilized the Risk of Bias In Non‐randomized Studies of Interventions (ROBINS‐I) tool.^[^ ^30^ ^]^ Cochrane developed this tool to evaluate potential biases associated with observational studies, which was implemented to evaluate the quality of nonrandomized studies of interventions.^[^ ^31^ ^]^ The quality assessments were conducted independently by SMG and SZM in duplicate. Disagreements were addressed by consensus with a third investigator (HM) (Table S2).

### Statistical analysis

For analyzing studies, this review employed RRs and 95% CIs as the effect estimate. In these studies, RR and HR were equivalent.^[^ ^32^ ^]^ Notably, if the incidence rate was <10% or the ORs spanned from 0.5 to 2.5, they are equivalent to RR; otherwise, OR was converted to RR employing the Zhang et al. approach.^[^ ^33^ ^]^

We utilized the random effects analysis to compute the combined RRs with 95% CIs for fracture risk linked to consuming the highest versus lowest amounts of fruit, as well as vegetables. Using the *I* ^2^ statistic, as well as the *Q* test, we evaluated heterogeneity between studies.^[^ ^34^ ^]^ We implemented subgroup analysis in accordance to region (Asia/Non‐Asia), age (<60/≥60 years), follow‐up duration (<10/≥10 years), sex (both/women), and controlling for confounders involving energy and alcohol intake, smoking, body mass index (BMI) and sex. In addition, meta‐regression analyses were conducted to identify possible origins of heterogeneity. To evaluate publication bias (when ≥10 studies were available), we performed Egger's and Begg's tests.^[^ ^35^, ^36^ ^]^ To assess the impact of each study on the final result, we implemented the influence analysis, which involved removing one study at a time and evaluating its relative influence on the summary estimate.

Dose‐dependent analysis was performed by applying the generalized least squares trend estimation method that was described by Greenland et al.^[^ ^37^, ^38^ ^]^ When using this method, various factors must be taken into account. These include the distribution of fracture events and total sample size or person‐years, as well as the controlled effect estimates throughout the different exposure categories. The median consumption is assigned as the corresponding RR or OR in each category. For studies without direct medians, the average point among the lower and upper limits was used to estimate approximate medians. We determined that the width of the open categories is equal to that of the adjacent category. The overall number of subjects, fracture events, or person‐years in every class of fruit and vegetables intake was divided by the number of classes in studies, in which the exposures were quantiles, but the numbers of subjects, fracture events, or person‐years were not stated.^[^ ^39^, ^40^ ^]^ We utilized a fixed effects model to merge the subgroup‐specific estimates from each study and included the combined effect estimates in the meta‐analysis when individual effect sizes were reported for different subgroups, such as gender. If the lowest category was not considered the reference group in the studies, we recalculated the effect sizes by assuming the lowest category as the reference.^[^ ^41^ ^]^ RRs or ORs for each 200‐g (one serving) per day increments in fruit and vegetables, only fruit, as well as only vegetables, were compiled utilizing random effects analysis. In cohort studies that provided the effect estimates for a certain increment in exposure, we multiplied the log effect estimate by the study's specific consumption of exposure and then exponentiated it. This allowed us to determine the effect size for each extra exposure serving.^[^ ^42^ ^]^

We also implemented a nonlinear dose‐dependent analysis applying limited cubic splines with three knots (10, 50, and 90 percentiles of the distribution).^[^ ^43^ ^]^ We examined the correlation within every group of published RRs and then combined the study‐certain estimates through a one‐stage mixed effects analysis.^[^ ^44^ ^]^ We used Stata to perform all our statistical analyses (Version 17; StataCorp, College Station, TX, USA). A statistically significant result was determined with a *p* value < 0.05.

### Grading the evidence

GRADE classifies evidence into four groups according to its certainty: “high,” “moderate,” “low,” and “very low.”^[^ ^45^ ^]^ Our certainty of evidence increased when we found large effect sizes or gradients in dose–response manner.

## Results

**Fig. 1:**
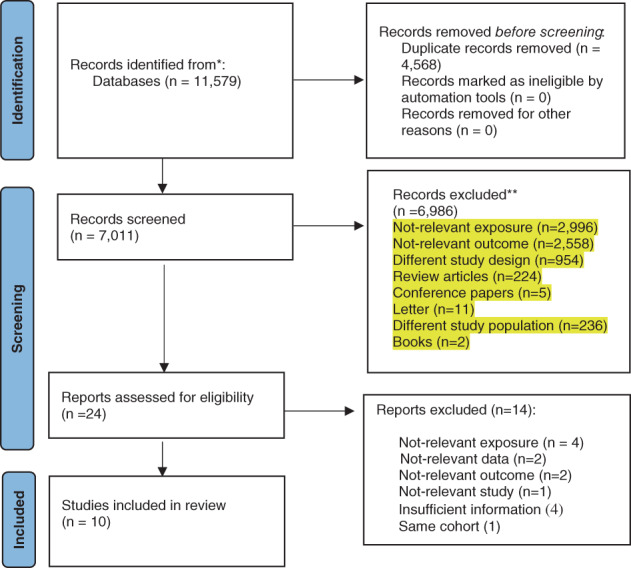
Literature search and study selection process.

A total of 11,579 records were found by literature search, of which 4568 were duplicates and 6986 were removed according to titles/abstracts reviewing (Fig. 1). After a thorough review of the full texts for the remaining studies, it was found that 14 studies had to be excluded based on the following justifications: (i) not‐relevant exposure (*n* = 4); (ii) not pertinent data (*n* = 2); (iii) not pertinent outcome (*n* = 2); (iv) inadequate data (*n* = 4); (v) not‐relevant study (*n* = 1); and (vi) duplicate (*n* = 1) (Table S3). In the end, 10 cohort studies involving 511,716 participants and 14,445 fracture events were chosen for analysis.^[^ ^15^, ^23^, ^24^, ^46^, ^47^, ^48^, ^49^, ^50^, ^51^, ^52^ ^]^

### Study characteristics

The study characteristics are outlined in detail in Table S4. Two studies were from Australia,^[^ ^47^, ^50^ ^]^ one from Sweden,^[^ ^23^ ^]^ one from Japan,^[^ ^49^ ^]^ one from France,^[^ ^48^ ^]^ one from Singapore,^[^ ^15^ ^]^ one from the UK,^[^ ^52^ ^]^ one from the United States,^[^ ^51^ ^]^ one from the United States and Europe,^[^ ^24^ ^]^ and one from 10 European countries.^[^ ^46^ ^]^ Follow‐up ranged from nine to 22.3 years, and the number of individuals ranged from 1429 to 142,018. Six studies were conducted on both sexes,^[^ ^15^, ^23^, ^24^, ^46^, ^48^, ^49^ ^]^ and the remaining four articles included only women.^[^ ^47^, ^50^, ^51^, ^52^ ^]^ Except for one study that assessed fruit and vegetables intake by applying weighed food records,^[^ ^49^ ^]^ others used food frequency questionnaires (FFQs).^[^ ^15^, ^23^, ^24^, ^46^, ^47^, ^48^, ^50^, ^51^, ^52^ ^]^ All entered cohort studies reported adjusted risk estimates; however, there were notable variations in the number and type of selected variables in maximum adjusted models. Of the entered 10 cohort studies, five had a serious risk of bias,^[^ ^24^, ^47^, ^49^, ^50^, ^51^ ^]^ while the remaining were judged as having a moderate risk of bias^[^ ^15^, ^23^, ^46^, ^48^, ^52^ ^]^ (Table S2).

### Total fruit and vegetable and fracture incidence

Five studies evaluated the relation among total fruit and vegetables and fracture incidence, involving 62,552 individuals and 2215 fracture events.^[^ ^23^, ^24^, ^51^, ^52^ ^]^ Pooled RR for the greatest against the lowest analysis was 0.82 (95% CI, 0.63 to 1.05; *I* ^2^ = 90.6%; *p*_heterogeneity_ <0.001) (Table 1, Fig. S1). We also found a nonstatistically significant association between total fruit and vegetables intake and any fracture incidence even after eliminating studies with serious risk of bias (RR 0.83; 95% CI, 0.55 to 1.24; *I* ^2^ = 95.6%; *p*_heterogeneity_ <0.001; *n* = 2). In the influence analysis, the summarized RR was significantly changed and revealed decreased fracture incidence (RR 0.72; 95% CI, 0.65 to 0.79) after the removal of the study by Webster et al.^[^ ^52^ ^]^

**Table 1:** Fruits and Vegetables Intake with Risk of Fracture

	Highest vs. lowest category meta‐analysis					Dose–response meta‐analysis				
Varia	Studies, *n*	RR (95% CI)	*I* ^2^, %	*p*_heterogeneity_	Dose, unit ser/day	Studies, *n*	RR (95% CI)	*I* ^2^, %	*p*_heterogeneity_	Certainty of evidence
Any fracture										
Total fruit and vegetable	5	0.82 (0.63, 1.05)	90.6%	<0.001	1	5	0.98 (0.95, 1.01)	89.4%	<0.001	Moderate
Fruit	6	0.97 (0.92, 1.03)	39.8%	0.140	1	3	0.96 (0.88, 1.05)	72.7%	0.026	Very low
Vegetable	6	0.84 (0.75, 0.95)	83.1%	<0.001	1	5	0.86 (0.77, 0.97)	84.7%	<0.001	Moderate
	2	0.82 (0.72, 0.92)^a^	65.3%	0.034						
Any fracture (after excluding studies with serious risk of bias)										
Total fruit and vegetable	2	0.83 (0.55, 1.24)	96.5%	<0.001	‐	‐	‐	‐	‐	‐
Fruit	4	0.96 (0.89, 1.05)	63.2%	0.043	‐	‐	‐	‐	‐	‐
Vegetable	3	0.88 (0.76, 1.03)	89.4%	<0.001	‐	‐	‐	‐	‐	‐

Abbreviations: Ser, serving; RR, relative risk; CI, confidence interval.

^a^ Cruciferous and green vegetables.

**Fig. 2:**
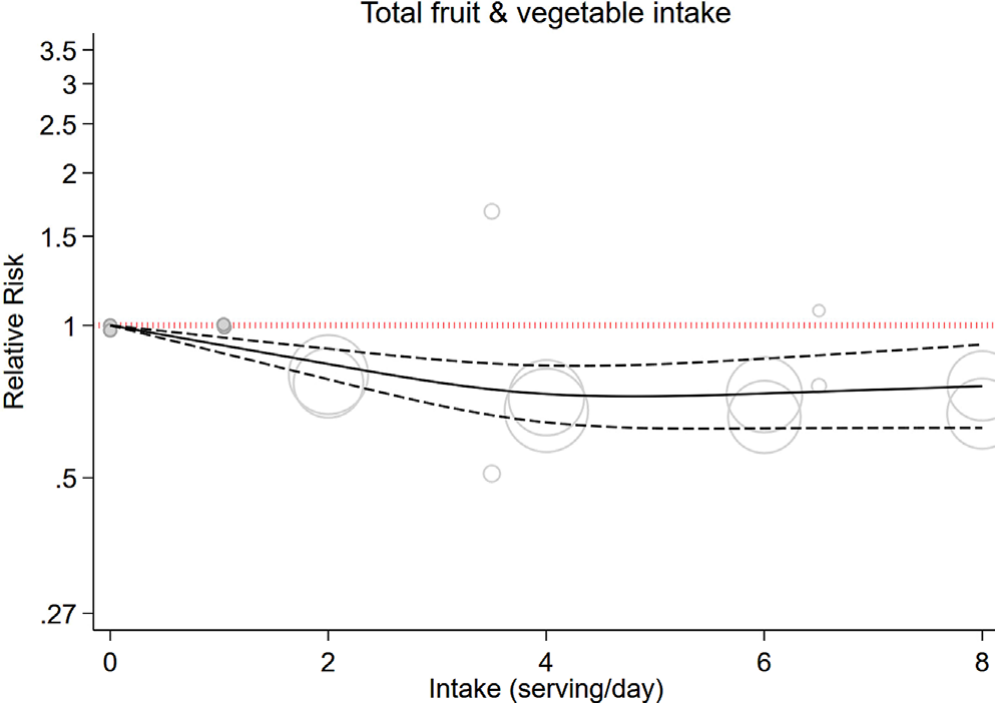
Dose–response associations of total fruit and vegetables intake and risk of any fracture in random‐effects model. Solid lines represent the relative risk of the association between total protein intake and fracture; dashed lines represent 95% CI. CI, confidence interval.

In dose‐dependent analysis, the pooled RR for per one serving (200 g) daily rise was 0.98 (95% CI, 0.95 to 1.01; *I* ^2^ = 89.4%; *p*_heterogeneity_ <0.001; *n* = 5; GRADE = moderate) (Table 1, Fig. S2). Moreover, a significant nonlinear relation was observed among total fruit and vegetables and incidence of fracture (*p*_nonlinearity_ <0.001; *n* = 5; Fig. 2).

### Fruit and fracture incidence

Six cohort studies involving 225,371 individuals with 3072 fracture events entered the highest versus lowest analysis.^[^ ^15^, ^46^, ^47^, ^48^, ^49^, ^52^ ^]^ The pooled RR indicated nonsignificant relation between fruit intake and any fracture incidence (RR 0.97; 95% CI, 0.92 to 1.03; *I* ^2^ = 39.8%; *p*_heterogeneity_ = 0.140; GRADE = very low) (Table 1, Fig. S3). This association was also nonsignificant even after eliminating studies with serious risk of bias (RR 0.96; 95% CI, 0.89 to 1.05; *I* ^2^ = 63.2%; *p*_heterogeneity_ = 0.043; *n* = 4). However, in the sensitivity analysis, the summarized RR was significantly changed and indicated lower fracture incidence (RR 0.93; 95% CI, 0.87 to 0.98) following the removal of the study by Webster et al.^[^ ^52^ ^]^

The RR remained the same in various regions and different age groups, except for cohort studies adjusted for energy intake, BMI, and sex, as well as studies that did not adjust for alcohol intake. Furthermore, fracture incidence was reduced significantly in studies that included both sexes (RR 0.93; 95% CI, 0.87 to 0.99; *I* ^2^ = 0%; *n* = 4), as well as studies with <10 years follow‐up (RR 0.91; 95% CI, 0.85 to 0.98; *I* ^2^ = 0%, *n* = 3) (Table S5).

In the linear dose‐dependent analysis, the pooled RR per one serving/day (200 g/day) increment was 0.96 (95% CI, 0.88 to 1.05; *I* ^2^ = 72.7%, *p*_heterogeneity_ = 0.026; *n* = 3; GRADE = very low) (Table 1, Fig. S4).

### Vegetable and fracture incidence

Six cohort studies examined the relation between vegetables intake and fracture incidence, involving 234,971, of whom 3272 faced fractures.^[^ ^15^, ^46^, ^47^, ^49^, ^50^, ^52^ ^]^ Summarized RR contrasting the extreme and lowest classes presented that vegetables intake was correlated with a 16% reduced fracture incidence (RR 0.84; 95% CI, 0.75 to 0.95; *I* ^2^ = 83.1%; *p*_heterogeneity_ <0.001) (Table 1, Fig. S5). This finding was similar when every study was omitted. There was also a significant relation among green and cruciferous vegetables intake and fracture risk at any site (RR 0.82; 95% CI, 0.72 to 0.92; *I* ^2^ = 65.13%; *p*_heterogeneity_ = 0.034) (Table 1). The subgroup analysis demonstrated significant findings in all subgroups (Table S6). However, we found a nonsignificant association among vegetables intake and fracture incidence after eliminating studies with serious risk of bias (RR 0.88; 95% CI, 0.76 to 1.03; *I* ^2^ = 89.4%; *p*_heterogeneity_ <0.001; *n* = 3).

According to the dose‐dependent analysis, consuming one serving of vegetables per day was linked to a reduced incidence of all types of fractures (RR 0.86; 95% CI, 0.77 to 0.97; *I* ^2^ = 84.7%; *p*_heterogeneity_ <0.001; *n* = 5; GRADE = moderate) (Table 1, Fig. S6).

## Discussion

Fracture is a considerable public health issue that affects millions of individuals globally and places a substantial strain on healthcare systems. Nutrition, specifically fruit and vegetables, is considered as a modifiable risk factor for preventing fractures.^[^ ^53^ ^]^ However, the proof of the relation among fruit and vegetables intake and fracture outcomes is inconsistent.^[^ ^54^ ^]^ Therefore, in this work we aimed to review the current literature on the role of fruit and vegetables in fracture prohibition and discuss the potential policy implications of promoting fruit and vegetables intake for bone health. We systematically reviewed available evidence on the possible protective effects of fruit, as well as vegetables intake and fracture incidence utilizing 10 cohort studies comprising 511,716 participants.

Quantitative synthesis revealed that only those with a greater intake of vegetables had a lower risk of any fracture. The dose‐dependent analysis also indicated a 14% decreased risk of any fracture with a greater intake of vegetables (GRADE = moderate quality). However, no statistically significant relation was obtained among total fruit and vegetables and fruit and any fracture in either high versus low analysis and dose‐dependent manner. Of note, in our review, after eliminating studies with serious risk of bias, we found nonsignificant associations between intake of total fruit and vegetables, only fruit or vegetables and risk of fracture at any site. As stated in the meta‐analysis of five observational studies involving 33,417 participants in 2016, consuming greater amount of vegetables alone was linked to a lower incidence of hip fractures (25%). However, no considerable relation was observed between consuming only fruit or both fruit and vegetables and hip fracture.^[^ ^25^ ^]^ On the other hand, a previous review indicated that a greater intake of fruit although not vegetables, was linked to reduced osteoporosis in postmenopausal women.^[^ ^55^ ^]^

Moreover, a review that compiled data from 31 observational studies proposed that higher compliance with a healthy dietary pattern was negatively linked to the risk of fracture.^[^ ^56^ ^]^ In 2019, a meta‐analysis discovered a connection between consuming fruit and vegetables and diminished hip fracture risk.^[^ ^26^ ^]^ This discrepancy with previous studies might be due to the fact that we considered all fracture events by including all related cohort studies. Also, a dose–response gradient for fruit and vegetables and the fracture incidence and using the GRADE tool substantially increase the certainty of evidence compared to previous meta‐analyses.

Eating fruit and vegetables is often recommended for a healthy diet; however, it is uncertain if they can prevent fractures. Fruit and vegetables contain alkaline ions such as calcium, magnesium, and potassium, which benefit our bone health. The link among magnesium and potassium consumption and healthy bone is suggested. Moreover, the role of calcium in maintaining bone health is well‐recorded.^[^ ^57^, ^58^, ^59^ ^]^ Fruit and vegetables may have a protective effect due to their great amount of vitamin K.^[^ ^60^ ^]^ Similarly, fruit and vegetables contain a high amount of antioxidants (e.g., carotenoids, carotene, and ascorbic acid), which may upregulate osteoblastic differentiation and downregulate osteoclastic differentiation and osteoclastogenesis via countering age‐related inflammation and oxidative stress.^[^ ^61^ ^]^ In addition, other nutrients (i.e., glutathione, tocotrienols, tocopherols, and polyphenols) may also reduce the risk of fractures.^[^ ^25^ ^]^ Additionally, a diet full of fruit and vegetables can lead to a lower dietary acid load. This has been linked to the inhibition of osteoclast function and the promotion of osteoblast activity, ultimately contributing to a reduction in the resorption of bone, as well as an elevation in bone formation.^[^ ^62^ ^]^

Furthermore, we found a significant relation among green and cruciferous vegetables intake and fracture risk at any site. It is important to note that, according to the geographical distribution of the studies, the availability and type of selected vegetables differ in each region. According to a study by Blekkenhorst et al.,^[^ ^47^ ^]^ the effect of cruciferous and allium vegetables in preventing fractures can be greater than other types of vegetables. Overall, green leafy vegetables^[^ ^63^ ^]^ and soybeans^[^ ^64^ ^]^ benefit bone health and can help prevent fractures.

Furthermore, some other actions, such as initiating supplements of vitamin D (at least 800 IU/day) and calcium (1200 mg/day) in older individuals (≥65 years) who have vertebral or hip fractures, especially in those who are unable to receive this amount from food sources might be helpful.^[^ ^65^ ^]^ However, the American College of Obstetricians and Gynecologists proposed that calcium and vitamin D supplementation might be less favorable in reducing risk of fracture.^[^ ^66^ ^]^ It is suggested that all postmenopausal patients who are 65 years or older undergo bone density screening. However, no policy exists regarding fruit and vegetables consumption to reduce fracture risk. In order to maintain a healthy diet, it is recommended for adults to consume 1.5–2‐cup equivalents of fruit and 2–3‐cup equivalents of vegetables daily. These guidelines are outlined in the Dietary Guidelines for Americans^[^ ^67^ ^]^; however, this is not specifically for reducing fracture risk. The level of evidence was very low for fruit consumption and fracture risk, translating into a weak recommendation for the general population to reduce the risk of fracture. However, the certainty of the evidence was moderate for vegetables, which could be interpreted as a stronger recommendation. However, since half of the included studies had a serious risk of bias (5/10), our findings should be interpreted with caution. Currently, there is no established policy on the recommended amount of fruit and vegetables consumption concerning the incidence of fractures in general adults. Larger longitudinal studies are necessary to enhance the level of evidence.

One of the major sources of bias among the included studies was confounding effects on the relationship among fruit and vegetables consumption and fracture incidence. In studies included in our meta‐analysis, various variables were controlled, including age, sex, race, anthropometric measures, medical and medication history, demographic variables, socioeconomic‐related variables, total energy intake, and other aspects of diet. However, the collection of adjusting variables was not the same, and other factors related to diet might not be considered, such as adjustment of major confounders (including vitamin D or calcium consumption), and they might be owing to the nonrandomized nature of the included cohorts; as a result, the possibility of residual confounders exists. Therefore, it is strongly suggested that future studies control for all known confounders based on available literature and also run sensitivity analyses to delineate the magnitude of unmeasured confounding needed to neutralize an effect altogether.^[^ ^68^ ^]^ Moreover, there is also a need to examine the relationship between greater intake of fruit and vegetables and fracture incidence, considering the different subtypes and preparation methods of fruits and vegetables because not all fruit and vegetables are nutritionally equal.

To our knowledge, this is the first review that quantitated the relationship between fruit and vegetables and any fracture risk in a dose‐dependent manner. We applied the multivariable‐adjusted models to reduce the confounding effects. Moreover, using the GRADE tool for assessing the quality of studies elevates our research's accuracy. Despite these strong points, some limitations warrant consideration. The certainty of the evidence was very low to moderate, mainly owing to the risk of bias in the entered studies and substantial heterogeneity. The impact of uncontrolled confoundings cannot be identified. Some studies had high heterogeneity, and the number of entered cohort studies was relatively small. Finally, considering the use of FFQ for dietary evaluation in most studies, the misclassification of individuals in relation to dietary intake should be considered.

## Conclusions

We discovered that individuals with greater consumption of vegetables had a lower risk of fracture with moderate certainty. Moreover, each serving/day increases the consumption of vegetables, leading to a 14% decrease in the risk of fracture. In contrast, nonstatistically significant relation was discovered among total fruit and vegetables, or only fruit and risk of overall fractures. Additional well‐designed intervention‐trials are highly essential to determine the link and underlying mechanisms.

## Author Contributions

All authors conceptualized and designed this review. SMG, SM, and AH conducted a literature search and collected the data. SZM performed an analysis. SZM, SMG, AA, SM, and AH wrote the first draft. HM and AA contributed to critically revising and interpreting the data. All authors approved the final manuscript.

### Peer Review

The peer review history for this article is available at https://www.webofscience.com/api/gateway/wos/peer-review/10.1002/jbm4.10840.

## Disclosures

All authors declare that they have no conflict of interest.

## Supporting information


**Data S1.** Supporting Information.

## Data Availability

The data that support the findings of this study are available on request from the corresponding author. The data are not publicly available due to privacy or ethical restrictions.
