# Supplementary material for: Fruit and Vegetable Consumption and the Risk of Bone Fracture: A Grading of Recommendations, Assessment, Development, and Evaluations (GRADE)‐Assessed Systematic Review and Dose–Response Meta‐Analysis
Source: JBMR Plus. 2023 Nov 10;7(12):e10840. doi: 10.1002/jbm4.10840 (PMC10731112; doi:10.1002/jbm4.10840)
Supplement: Supplementary file 1 — Data S1. Supporting Information. [file JBM4-7-e10840-s001.doc]

**Fruit and vegetable consumption and the risk of bone fracture: A GRADE-assessed systematic review and dose-response meta-analysis**

Online Supplementary Material Supporting Materials including Supplementary Tables 1-7, Supplementary Figure 1-6, and Supplementary references

| **Database**  **12/14/2022** | **key terms and the queries** |
| --- | --- |
| PubMed  N=3286 | **#1**((Fruit OR vegetable OR fruits OR vegetables OR fruit*) OR (Fruit OR vegetable OR fruits OR vegetables OR fruit*[MeSH Terms]))  **#2** (bone [Title/Abstract] OR "bone fracture"[Title/Abstract] OR fracture [Title/Abstract] OR "osteoporotic fracture"[Title/Abstract] OR "broken bone"[Title/Abstract] OR "bone mineral density"[Title/Abstract] OR BMD[Title/Abstract] OR "bone health"[Title/Abstract] OR osteoporosis [Title/Abstract] OR “Bone Density” [Title/Abstract]) OR ("Fractures, Bone" OR "Bone and Bones" OR "Osteoporotic Fractures" OR "Bone Density" OR Osteoporosis [MeSH Terms])  #1 AND #2 |
| Web of Science (ISI)  N=3123 | **#1** TOPIC: (Fruit OR vegetable OR fruits OR vegetables OR fruit*)  **#2** TOPIC: (bone OR “bone fracture” OR fracture OR "osteoporotic fracture” OR “broken bone” OR “bone mineral density” OR BMD OR “bone mass density” OR osteoporosis OR “bone health” OR “Bone and Bones” )  #1 AND #2 |
| Scopus  N=5170 | **#1**( TITLE-ABS-KEY ( fruit )  OR  TITLE-ABS-KEY ( vegetable )  OR  TITLE-ABS-KEY ( fruits )  OR  TITLE-ABS-KEY ( vegetables ) )  **#2** ( TITLE-ABS-KEY ( bone )  OR  TITLE-ABS-KEY ( "bone fracture" )  OR  TITLE-ABS-KEY ( fracture )  OR  TITLE-ABS-KEY ( "osteoporotic fracture" )  OR  TITLE-ABS-KEY ( "broken bone" )  OR  TITLE-ABS-KEY ( "bone mineral density" )  OR  TITLE-ABS-KEY ( bmd )  OR  TITLE-ABS-KEY ( "bone mass density" )  OR  TITLE-ABS-KEY ( osteoporosis )  OR  TITLE-ABS-KEY ( "bone health" )  OR  TITLE-ABS-KEY ( "Bone and Bones" ) )  #1 AND #2 |

**Supplementary Table 1.** Search strategies including the key terms and the queries for each database.

**Supplementary Table 2.** ROBINS-I judgement for each domain and overall.

| **Author (year, Country)** | **Bias of confounding** | **Bias in selection of participants into the study** | **Bias due to exposure assessment** | **Bias due to misclassification during follow-up** | **Bias of missing data** | **Bias in measurement of outcomes** | **Bias in selection of the reported result** | **Overall judgement** |
| --- | --- | --- | --- | --- | --- | --- | --- | --- |
| Blekkenhorst et al. (2017, Australia) (1) | Serious | Low | Moderate | Low | Low | Low | Low | Serious |
| Byberg et al. (2014, Sweden) (2) | Moderate | Low | Moderate | Moderate | Moderate | Low | Low | Moderate |
| Benetou et al. (2016, Europe & USA) (3) | Serious | Low | Moderate | Moderate | Low | Low | Low | Serious |
| Benetou et al. (2012, 10 European countries) (4) | Moderate | Low | Moderate | Moderate | Low | Low | Low | Moderate |
| Dai,et al. (2014, Singapore) (5) | Moderate | Low | Moderate | Moderate | Moderate | Moderate | Low | Moderate |
| Feart et al. (2013, France) (6) | Moderate | Low | Moderate | Low | Low | Serious | Low | Moderate |
| Sim et al. (2018, Australia) (7) | Serious | Low | Moderate | Moderate | Low | Low | Low | Serious |
| Thorpe et al. (2007, USA) (8) | Serious | Low | Moderate | Moderate | Moderate | Moderate | Low | Serious |
| Saito et al. (2022, Japan) (9) | Serious | Low | Serious | Moderate | Moderate | Moderate | Low | Serious |
| Webster et al. (2022, UK) (10) | Moderate | Low | Moderate | Moderate | Low | Low | Low | Moderate |

| **Supplemental Table 3**. Reason for exclusion of retrieved articles. | |
| --- | --- |
| **References** | **Reason for exclusion** |
| 1. Zhang, X., Shu, X. O., Li, H., Yang, G., Li, Q., Gao, Y. T., & Zheng, W. (2005). Prospective cohort study of soy food consumption and risk of bone fracture among postmenopausal women. Archives of internal medicine, 165(16), 1890-1895.‏ | not-relevant exposure |
| 1. Lim, Y. S., Lee, S. W., Tserendejid, Z., Jeong, S. Y., Go, G., & Park, H. R. (2015). Prevalence of osteoporosis according to nutrient and food group intake levels in Korean postmenopausal women: using the 2010 Korea National Health and Nutrition Examination Survey Data. Nutrition research and practice, 9(5), 539-546.‏ | not-relevant data |
| 1. Kardinaal, A. F. M., Morton, M. S., Brüggemann-Rotgans, I. E. M., & Van Beresteijn, E. C. H. (1998). Phyto-oestrogen excretion and rate of bone loss in postmenopausal women. European Journal of Clinical Nutrition, 52(11), 850-855.‏ | not-relevant outcome |
| 1. Women’s Health Initiative Investigators McTiernan Anne amctiern@ fhcrc. org Wactawski-Wende Jean Wu LieLing Rodabough Rebecca J Watts Nelson B Tylavsky Frances Freeman Ruth Hendrix Susan Jackson Rebecca. (2009). Low-fat, increased fruit, vegetable, and grain dietary pattern, fractures, and bone mineral density: the Women’s Health Initiative Dietary Modification Trial. The American journal of clinical nutrition, 89(6), 1864-1876.‏ | not-relevant study |
| 1. Lin, C. H., Chen, K. H., Chen, C. M., Chang, C. H., Huang, T. J., Hsu, H. C., & Huang, S. Y. (2016). Low vegetable intake increases the risk of fall-related fragility fracture in postmenopausal Taiwanese women, a prospective pilot study in the community. biomedical journal, 39(3), 214-222.‏ | insufficient information |
| 1. Weikert, C., Walter, D., Hoffmann, K., Kroke, A., Bergmann, M. M., & Boeing, H. (2005). The relation between dietary protein, calcium and bone health in women: results from the EPIC-Potsdam cohort. Annals of nutrition and metabolism, 49(5), 312-318.‏ | not-relevant outcome |
| 1. Petridou, E., Karpathios, T., Dessypris, N., Simou, E., & Trichopoulos, D. (1997). The role of dairy products and non alcoholic beverages in bone fractures among schoolage children. Scandinavian journal of social medicine, 25(2), 119-125.‏ | not-relevant exposure |
| 1. Farsijani, S., Cauley, J. A., Peddada, S. D., Langsetmo, L., Shikany, J. M., Orwoll, E. S., ... & Newman, A. B. (2022). Relation Between Dietary Protein Intake and Gut Microbiome Composition in Community-Dwelling Older Men: Findings from the Osteoporotic Fractures in Men Study (MrOS). The Journal of Nutrition, 152(12), 2877-2887.‏ | not-relevant exposure |
| 1. Ballesteros, J. M., Struijk, E. A., Rodríguez-Artalejo, F., & López-García, E. (2020). Mediterranean diet and risk of falling in community-dwelling older adults. Clinical nutrition, 39(1), 276-281.‏ | not-relevant data |
| 1. Lanyan, A., Marques-Vidal, P., Gonzalez-Rodriguez, E., Hans, D., & Lamy, O. (2020). Postmenopausal women with osteoporosis consume high amounts of vegetables but insufficient dairy products and calcium to benefit from their virtues: the CoLaus/OsteoLaus cohort. Osteoporosis international, 31, 875-886.‏ | insufficient information |
| 1. Nguyen HH, Wu F, Oddy WH, Wills K, Winzenberg T, Jones G: Associations between dietary patterns and osteoporosis-related outcomes in older adults: a longitudinal study. European Journal of Clinical Nutrition 2021, 75:792-800. | insufficient information |
| 1. Michaelsson K, Wolk A, Lemming EW, Melhus H, Byberg L: Intake of milk or fermented milk combined with fruit and vegetable consumption in relation to hip fracture rates: a cohort study of Swedish women. *Journal of Bone and Mineral Research* 2018, 33:449-457. | insufficient information |
| 1. Dai Z, Butler LM, van Dam RM, Ang L-W, Yuan J-M, Koh W-P: Adherence to a vegetable-fruit-soy dietary pattern or the Alternative Healthy Eating Index is associated with lower hip fracture risk among Singapore Chinese. The Journal of nutrition 2014, 144:511-518. | not-relevant exposure |
| 1. Benetou V, Orfanos P, Zylis D, Sieri S, Contiero P, Tumino R, Giurdanella M, Peeters P, Linseisen J, Nieters A: Diet and hip fractures among elderly Europeans in the EPIC cohort. European journal of clinical nutrition 2011, 65:132-139. | Same cohort |

**Supplementary Table 4**. Characteristics of included studies.

| **Author (year, Country), ref** | **Study name** | **Age range, sex (men/women/both)** | **Follow-up, years** | **Total number of participants/Cases** | **Exposure**  **type** | **Comparison**  **(lowest vs. highest)** | **Exposure assessment** | **Type of fracture** | **Outcome assessment** | **Adjustments** |
| --- | --- | --- | --- | --- | --- | --- | --- | --- | --- | --- |
| Blekkenhorst et al. (2017, Australia) (1) | PLSAW | ≥70 years,  Older woman | 14.5 | 1,468/415 | Fruit  Vegetable | <1 serving/day compared with ≥2 serving/day | Validated self-administered, semiquantitative FFQ | Any fracture | Hospital Morbidity Data Collection, linked via the Western Australian Data Linkage System | 1,4,5,6,7,8,15,16,17,18,27&59 |
| Byberg et al.  (2014, Sweden) (2) | COSM & SMC | 45-83 years,  Both | 14.2 | 75,591/3,644 | Fruit and Vegetable | <1 serving/day compared with >7 serving/day | Validated 96-item FFQ-Interview | Hip fracture | ICD-10 codes S72.0, S72.1, or S72.2 | 1,2,4,27,15,18,16,5,12,17,20 |
| Benetou et al. (2016, Europe & USA) (3) | CHANCES | ≤60 years,  Both | 13.45 | 142,018/5552 | Fruit and Vegetable | ≤1 serving/day compared with >7 serving/day | Validated 84- & 260-item FFQ-self or interviewer-administered | Hip fracture | ICD-10 codes S72.0, S72.1, or S72.2 | 1,4,5,15,16,17,18,33,43&46 |
| Benetou et al. (2012, 10 European countries) (4) | EPIC study | ≤50 years,  Both | 9 | 188,795 /802 | Fruit  Vegetable | Continues (g/day) | Validated FFQ-self or interviewer-administered or diet history questionnaire | Hip fracture | Active follow-up methods or record linkage with hospital discharge records or through hip and radius fracture registries | 1,2,5,15,4,18,17,27,28,26 |
| Dai,et al. (2014, Singapore) (5) | Singapore Chinese Health Study | 45-74,  Both | 9.9 | 63,257/1,630 | Fruit  Vegetable | Q1 vs. Q4 | Validated 165‐item semiquantitative FFQ-face-to-face interview | Hip fracture | Record linkage analysis with hospital discharge databases of the MediClaim System | 1,4,5,17,15,18,27,33 |
| Feart et al. (2013, France) (6) | Three-City study | >65,  Both | 8 | 1,482/155 | Fruit  Vegetable | Continues (servings/week) | Notsemiquantitative 20-item FFQ & 24-h dietary recall-interview | Any fracture | Self-reported | 1,2,18,17,5,4,6 |
| Sim et al. (2018, Australia) (7) | PLSAW | ≥70 years,  Older women | 14.5 | 1,429/404 | Vegetable | ≤3 servings/day compared with ≥5 serving/day | Validated semi-quantitative FFQ-self-administered | Any fracture | Western Australian Data Linkage System | 1,4,5,6,7,8,15,16,17,18,27&59 |
| Thorpe et al. (2007, USA) (8) | Adventist Health Study | >35 years**,** Peri- and post-menopausal women | 25 | 1,865/216 | Fruit and Vegetable | <2 serving/day compared with >5 serving/day | Validated 65-item semi-quantitative FFQ-interview | Wrist fracture | Record linkage | 4,5,18,33,47&48 |
| Saito et al. (2022, Japan) (9) | NIPPON DATA | ≥30 years, Both | 12.1 | 9,133/805 | Fruit  Vegetable | Continues (fruit: 49.3g/100kcal, vegetable: 46.4 g/1000kcal) | Weighed food records on three consecutive representative days-interview | Lower limb fractures | Home-visit interviews | Multivariable adjusted |
| Webster et al. (2022, UK) (10) | UKWCS | 35-69 yreas,  Middle-aged woman | 22.3 | 26,318 /822 | Fruit  Vegetable  Fruit and Vegetable | Continues (80 g/day) | Validated a self-administered 217-item FFQ | Hip fracture | ICD-9 code 820, ICD-10 codes S72.0-72.2 | 6,27,26,28,18,15,16,20 |

**Abbreviations**: CHANCES, The Consortium on Health and Ageing: Network of Cohorts in Europe and the United States; COSM, population-based Cohort of Swedish Men; EPIC, European Prospective Investigation into Cancer and Nutrition; FFQ, Food Frequency Questionnaire; ICD, International Classification of Diseases; NIPPON DATA, National Integrated Project for Prospective Observation of Non-communicable Disease And its Trends in the Aged; PLSAW, Perth Longitudinal Study of Aging in Women; Q, quartiles; ref, reference; RR: Risk Ratio; SMC, Swedish Mammography Cohort; UKWCS, UK Women's Cohort Study.

^a^1.age, 2. sex, 3. race, 4. body mass index, 5. educational status or highest academic degree, 6. marital status, 7. occupational position, 8. income, 9. residence, 10. Size of residential area 11. Wealth or household wealth, 12. living alone, 13. family arrangement, 14. census track income data 15. Tobacco or smoking, 16. alcohol drinking, 17. energy intake, 18. physical activity level, 19. fruit and vegetable intake, 20. dietary supplement use 21. cognition, 22. depressed mood or depression, 23. medical history, 24. self-rated health, 25. history of chronic disease, 26. cardiovascular disease, 27. diabetes mellitus, 28. cancer, 29. asthma or chronic bronchitis, 30. osteo-muscular disease, 31. hyperlipidemia, 32. Number of drug treatment, 33. postmenopausal hormone use, 34. aspirin, 35. antihypertensive medications, 36. lipid lowering medications, 37. diabetes medication, 38. insulin, 39. Mini-mental state examination score, 40. modified trichopoulou index, 41. Charlson co-morbidity index, 42. modified Alternate Healthy Eating Index that does not include fruits and vegetables, 43. number of morbidities at age 50, 44. wave of inclusion, 45. All other health behaviors, 46. Height, 47. vegetable, 58. Cheese, 59. calcium.

**Supplementary Table 5.** Subgroup analyses of fruit intake and risk of fracture (Highest vs. lowest category meta-analysis).

| **Sub-grouped by** | **Number of effect sizes** | **Relative Risk (95%CI)** | **I^2^ (%)** | **P for heterogeneity** | **P for between**  **subgroup heterogeneity** |
| --- | --- | --- | --- | --- | --- |
| All studies | 6 | 0.97 (0.92, 1.03) | 39.8% | 0.14 | - |
| Region | | | | | **0.83** |
| Asia | 2 | 0.98 (0.89, 1.08) | 0.0% | 0.92 |  |
| Non-Asia | 4 | 0.96 (0.88, 1.05) | 62.9% | 0.04 |  |
| Age | | | | | **0.75** |
| < 60 | 3 | 0.96 (0.87, 1.05) | 75.5% | 0.02 |  |
| ≥ 60 | 3 | 0.98 (0.88, 1.09) | 0.0% | 0.98 |  |
| Follow-up duration | | | | | **0.01** |
| < 10 years | 3 | 0.91 (0.85, 0.98) | 0.0% | 0.54 |  |
| ≥ 10 years | 3 | 1.01 (0.98, 1.03) | 0.0% | 0.85 |  |
| Sex |  |  |  |  | **0.01** |
| Women | 2 | 1.01 (0.99, 1.03) | 0.0% | 0.75 |  |
| Both | 4 | 0.93 (0.87, 0.99) | 0.0% | 0.53 |  |
| Adjustment for confounders | | | | | |
| Energy intake | | | | | **0.01** |
| Yes | 5 | 0.93 (0.88, 0.99) | 0.0% | 0.67 |  |
| No | 1 | 1.01 (0.99, 1.04) | - | - |  |
| Physical activity | | | | | **-** |
| Yes | 6 | 0.97 (0.92, 1.03) | 39.8% | 0.14 |  |
| No | 0 | - | - | - |  |
| Alcohol intake | | | | | **0.01** |
| Yes | 3 | 1.01 (0.98, 1.03) | 0.0% | 0.85 |  |
| No | 3 | 0.91 (0.85, 0.98) | 0.0% | 0.54 |  |
| Smoking status | | | | | **0.81** |
| Yes | 5 | 0.97 (0.91, 1.03) | 51.8% | 0.08 |  |
| No | 1 | 1.01 (0.72, 1.42) | - | - |  |
| BMI | | | | | **0.01** |
| Yes | 5 | 0.93 (0.88, 0.99) | 0.0% | 0.67 |  |
| No | 1 | 1.01 (0.99, 1.04) | - | - |  |
| Sex | | | | | **0.01** |
| Yes | 2 | 0.90 (0.83, 0.97) | 0.0% | 0.48 |  |
| No | 4 | 1.01 (0.98, 1.03) | 0.0% | 0.91 |  |

CI, confidence intervals; ICD, International Classification of Diseases; BMI, body mass index.

**Supplementary Table 6.** Subgroup analyses of vegetable intake and risk of fracture (Highest vs. lowest category meta-analysis).

| **Sub-grouped by** | **Number of effect sizes** | **Relative Risk (95%CI)** | **I^2^ (%)** | **P for heterogeneity** | **P for between**  **subgroup heterogeneity** |
| --- | --- | --- | --- | --- | --- |
| All studies | 6 | 0.84 (0.75, 0.95) | 83.1% | <0.001 | - |
| Region | | | | | **0.35** |
| Asia | 2 | 0.80 (0.73, 0.89) | 0.0% | 0.41 |  |
| Non-Asia | 4 | 0.87 (0.76, 1.01) | 83% | 0.001 |  |
| Age | | | | | **0.22** |
| < 60 | 4 | 0.87 (0.76, 0.99) | 87.3% | <0.001 |  |
| ≥ 60 | 2 | 0.76 (0.63, 0.91) | 0.0% | 0.72 |  |
| Follow-up duration | | | | | **0.72** |
| < 10 years | 2 | 0.83 (0.74, 0.93) | 40.8% | 0.19 |  |
| ≥ 10 years | 4 | 0.86 (0.73, 1.01) | 82.1% | 0.001 |  |
| Sex |  |  |  |  | **0.84** |
| Women | 3 | 0.86 (0.68, 1.08) | 77.9% | 0.01 |  |
| Both | 3 | 0.84 (0.78, 0.89) | 0.0% | 0.43 |  |
| Adjustment for confounders | | | | | |
| Energy intake | | | | | **0.45** |
| Yes | 4 | 0.83 (0.78, 0.88) | 0.0% | 0.46 |  |
| No | 2 | 0.91 (0.71, 1.17) | 76.4% | 0.04 |  |
| Physical activity | | | | | **0.52** |
| Yes | 5 | 0.85 (0.75, 0.97) | 85.2% | <0.001 |  |
| No | 1 | 0.78 0.61, 0.99) | - | - |  |
| Alcohol intake | | | | | **0.60** |
| Yes | 3 | 0.88 (0.73, 1.06) | 84% | 0.002 |  |
| No | 3 | 0.83 (0.77, 0.90) | 0.0% | 0.37 |  |
| Smoking status | | | | | **0.52** |
| Yes | 5 | 0.85 (0.75, 0.97) | 85.2% | <0.001 |  |
| No | 1 | 0.78 (0.61, 0.99) | - | - |  |
| BMI | | | | | **0.46** |
| Yes | 4 | 0.83 (0.78, 0.88) | 0.0% | 0.46 |  |
| No | 2 | 0.91 (0.71, 1.17) | 76.4% | 0.04 |  |
| Sex | | | | | **0.73** |
| Yes | 1 | 0.86 (0.79, 0.94) | - | - |  |
| No | 5 | 0.83 (0.71, 0.97) | 83.5% | <0.001 |  |

CI, confidence intervals; ICD, International Classification of Diseases; BMI, body mass index.

**Supplementary Table 7.** GRADE evidence table for the association of total fruit& vegetable with risk of any fracture.

| **Certainty assessment** | | | | | | | **№ of patients** | | **Effect** | | **Certainty** |
| --- | --- | --- | --- | --- | --- | --- | --- | --- | --- | --- | --- |
| **№ of studies** | **Study design** | **Risk of bias** | **Inconsistency** | **Indirectness** | **Imprecision** | **Other considerations** | **case** | **participants** | **Relative (95% CI)** | **Absolute (95% CI)** |  |
| 5 | observational studies | serious^a^ | serious^b^ | not serious | not serious^c^ | dose response gradient | 2170/62552 (3.5%) | 62552 | **RR 0.98** (0.95 to 1.01) | **1 fewer per 1,000** (from 1 fewer to 1 fewer) | ⨁⨁⨁◯ Moderate |
| 3 | observational studies | serious^a^ | serious^d^ | serious^e^ | serious^f^ | dose response gradient | 1761/215604 (0.8%) | 215604 | **RR 0.96** (0.88 to 1.05) | **1 fewer per 1,000** (from 1 fewer to 1 fewer) | ⨁◯◯◯ Very low |
| 5 | observational studies | serious^a^ | serious^g^ | not serious | not serious^h^ | dose response gradient | 2467/231837 (1.1%) | 231837 | **RR 0.86** (0.77 to 0.97) | **1 fewer per 1,000** (from 1 fewer to 1 fewer) | ⨁⨁⨁◯ Moderate |

**CI:** confidence interval; **RR:** risk ratio

#### Explanations

a. Downgraded since most studies judged as serious risk of bias based on ROBINS-I were included in the meta-analysis and residual confounding cannot be ruled out. Downgraded.

b. Serious inconsistency since I2 = 89%, Phet <0.001. Downgraded

c. No serious imprecision since the lower and upper limits were between 0.9 and 1.1. Not downgraded

d. Serious inconsistency since I2 = 72%, Phet =0.026. Downgraded

e. Serious indirectness since only three studies were available. Downgraded.

f. Serious imprecision since 95% included the null value and the lower bound was <0.90. Downgraded.

g. Serious inconsistency since I2 = 84%, Phet <0.001. Downgraded

h. No serious imprecision since 95%CI in the linear dose-response meta-analyses included a significant effect. Not downgraded.

**Supplementary Figure 1.** Forest plots for the highest versus lowest analysis. The association between total fruit and vegetable intake and risk of any fracture. Diamonds represent pooled estimates from random-effects analysis. RR: Risk ratio, CI: confidence interval.

**Supplementary Figure 2.** Forest plots for the linear analysis. The association between total fruit and vegetable intake and risk of any fracture. Diamonds represent pooled estimates from random-effects analysis. RR: Risk ratio, CI: confidence interval.

**Supplementary Figure 3.** Forest plots for the highest versus lowest analysis. The association between fruit intake and risk of any fracture. Diamonds represent pooled estimates from random-effects analysis. RR: Risk ratio, CI: confidence interval.

**Supplementary Figure 4.** Forest plots for the linear analysis. The association between fruit intake and risk of any fracture. Diamonds represent pooled estimates from random-effects analysis. RR: Risk ratio, CI: confidence interval.

**Supplementary Figure 5.** Forest plots for the highest versus lowest analysis. The association between vegetable intake and risk of any fracture. Diamonds represent pooled estimates from random-effects analysis. RR: Risk ratio, CI: confidence interval.

**Supplementary Figure 6.** Forest plots for the linear analysis. The association between vegetable intake and risk of any fracture. Diamonds represent pooled estimates from random-effects analysis. RR: Risk ratio, CI: confidence interval.

**Supplementary References**

1. Blekkenhorst LC, Hodgson JM, Lewis JR, Devine A, Woodman RJ, Lim WH, et al. Vegetable and fruit intake and fracture-related hospitalisations: a prospective study of older women. Nutrients. 2017;9(5):511.

2. Byberg L, Bellavia A, Orsini N, Wolk A, Michaëlsson K. Fruit and vegetable intake and risk of hip fracture: a cohort study of Swedish men and women. Journal of Bone and Mineral Research. 2015;30(6):976-84.

3. Benetou V, Orfanos P, Feskanich D, Michaëlsson K, Pettersson‐Kymmer U, Eriksson S, et al. Fruit and vegetable intake and hip fracture incidence in older men and women: the CHANCES project. Journal of Bone and Mineral Research. 2016;31(9):1743-52.

4. Benetou V, Orfanos P, Pettersson-Kymmer U, Bergström U, Svensson O, Johansson I, et al. Mediterranean diet and incidence of hip fractures in a European cohort. Osteoporos Int. 2013;24(5):1587-98.

5. Dai Z, Wang R, Ang LW, Low YL, Yuan JM, Koh WP. Protective effects of dietary carotenoids on risk of hip fracture in men: the Singapore Chinese Health Study. Journal of Bone and Mineral Research. 2014;29(2):408-17.

6. Feart C, Lorrain S, Ginder Coupez V, Samieri C, Letenneur L, Paineau D, et al. Adherence to a Mediterranean diet and risk of fractures in French older persons. Osteoporosis International. 2013;24:3031-41.

7. Sim M, Blekkenhorst LC, Lewis JR, Bondonno CP, Devine A, Zhu K, et al. Vegetable diversity, injurious falls, and fracture risk in older women: a prospective cohort study. Nutrients. 2018;10(8):1081.

8. Thorpe DL, Knutsen SF, Beeson WL, Rajaram S, Fraser GE. Effects of meat consumption and vegetarian diet on risk of wrist fracture over 25 years in a cohort of peri-and postmenopausal women. Public health nutrition. 2008;11(6):564-72.

9. Saito Y, Miura K, Arima H, Hayakawa T, Takashima N, Kita Y, et al. Predictors of lower limb fractures in general Japanese: NIPPON DATA90. PLoS One. 2022;17(2):e0261716.

10. Webster J, Greenwood DC, Cade JE. Foods, nutrients and hip fracture risk: A prospective study of middle-aged women. Clin Nutr. 2022;41(12):2825-32.
